# Functional Polyimide-Based Electrospun Fibers for Biomedical Application

**DOI:** 10.3390/ma12193201

**Published:** 2019-09-29

**Authors:** Diana Serbezeanu, Tăchiță Vlad-Bubulac, Daniela Rusu, Grațiela Grădișteanu Pircalabioru, Iuliana Samoilă, Sorina Dinescu, Magdalena Aflori

**Affiliations:** 1 “Petru Poni” Institute of Macromolecular Chemistry, Aleea Grigore Ghica Voda, 41A, 700487 Iasi, Romania; tvladb@icmpp.ro (T.V.-B.); rusu.daniela@icmpp.ro (D.R.); 2 Sanimed International IMPEX SRL, Sos. București Măgurele 70F, 051434 Bucharest, Romania; gratiela87@gmail.com; 3 Department of Biochemistry and Molecular Biology, University of Bucharest, 91-95 Splaiul Independentei, 050095 Bucharest, Romania; iuliana.samoila@bio.unibuc.ro (I.S.); sorina.dinescu@bio.unibuc.ro (S.D.)

**Keywords:** electrospun polyimide fibers, dermal fibroblasts, MTT test, Live-Dead test, antimicrobial properties, anti-biofilm activity

## Abstract

The current study focuses on the application of cytotoxicity tests upon one membrane matrix based on electrospun polyimide fibers, appealing for biomedical application, such as scaffolds for cell growth, patches or meshes for wound healing, etc. Assays were performed in order to determine the viability and proliferation of L929 murine fibroblasts after they were kept in direct contact with the studied electrospun polyimide fibers. Increased cell viability and proliferation were detected for cells seeded on electrospun polyimide fibers membrane, in comparison with the control system, either after two or six days of evaluation. The number of live cells was higher on the studied material compared to the control, after two and six days of cell seeding. The tendency of the cells to proliferate on the electrospun polyimide fibers was revealed by confocal microscopy. The morphological stability of electrospun polyimide membrane was evaluated by SEM observation, after immersion of the samples in phosphate buffer saline solution (PBS, 7.4 at 37 °C) at various time intervals. Additionally, the easy production of electrospun polyimide fibers can facilitate the development of these types of matrices into specific biomedical applications in the future.

## 1. Introduction

Over the past few years, new worldwide public health challenges, such as antimicrobial resistance and biofilm formation were introduced into the agenda of some scientific communities, thus, governing the development of novel bioactive molecules capable of fighting against multidrug resistant bacteria [1,2,3]. Moreover, some studies revealed the approval of bio-medicinal herbal extracts or infusions of plants to deal with infectious pathogens in various diseases, opening the door for new development steps in medicine branches, such as skin regeneration, wound healing, tissue dressing, etc. For these types of applications, few groups used bioactive electrospun fibers as support matrix for their herbal extracts, in order to accelerate the healing/repair process of the treated tissue [4,5,6,7,8].

Electrospinning is an inexpensive and simple process to produce fibers using electrostatic force between surface charges to continuously draw nanofibers using a viscoelastic liquid. In the development of electrospun polymer fibers, diameter, and morphology can be controlled by a number of experimental parameters such as: Solution viscosity, charge density, surface tension, polymer molecular weight, dielectric constant, flow rate applied voltage, and tip-to-collector. Thus, nanofibers obtained using this technique exhibit a very large surface area, nanoscale structure and porosity, properties which recommend these electrospun fibers for a wide spectrum of application such as: environmental protection, water purification, air filtration, smart textiles, encapsulation of bioactive species, drug delivery, tissue engineering, and regenerative medicine [9,10,11,12,13,14,15].

An important class of high-performance polymers having excellent thermal, electrical, and mechanical properties, as well as remarkable chemical resistance is represented in the literature by aromatic polyimides [16,17,18,19]. Due to their outstanding performances, polyimides have been found very useful for numerous applications, for example, in ultra-large scale integrate circuit, multichip module packaging, printed circuit board fabrication [20,21,22], and lately they came into attention of the specialists from the biomedical field. For example, polyimide-based materials have been used for the construction of electrodes in biomedical microdevices [23,24] or for the manufacture of fibrous membranes operating as oxygenators in intravenous or intrathoracic devices, helping the optimal function of the lungs [25]. The biosafety of polyimides have been evidenced when this type of material was used for encapsulation of biosensors and more recently as a substrate for neuronal or ocular implants. Thus, *in vitro* tests indicated that polyimide implants were biocompatible, causing only moderate coagulation, minor cytotoxicity, and hemolysis [26], while supporting cell adhesion and cell growth in the presence of fibroblasts [27]. *In vivo*, polyimide implants showed good tissue integration and induced a very weak foreign body reaction, limited to a small area around the implants after an extended period of time [28]. Van Vlierberghe et al. have shown that following chemical modification the polyimides maintain their biocompatibility [29].

To date, electrospun nanofibers matrices appealing for the pharmaceutical industry have been studied, but to the best of our knowledge, reports about biological properties of electrospun fibers based on polyimide are rather rare in the literature. Electrospun polyimide fibers combine the advantages of polyimides, as a high-performance polymer class, with electrospinning process, as a tool for production of nanofiber membranes showing excellent properties, which attracted much attention from scientific worldwide communities [30,31,32,33,34,35,36]. Electrospun polyimide fibers have several characteristics such as large surface area to volume ratio, and a porous structure, due to the nanoscale network such as morphology. As the porosity of a scaffold is higher, the more homogeneous will be the cell distribution and interconnection for an engineered tissue [37,38]. These properties recommend polyimides nanofibers for a variety of biomedical applications such as drug delivery, medical implants, tissue engineering, and body–implant interphases [39]. Up to now, considerable efforts have been devoted to various electrospun polyimide fibers [30,40,41,42]. However, nearly no effort has been given to the development of electrospun polyimide fibers used in medical domain.

This work focuses on the synthesis of one polyimide bearing hydroxyl functional groups (DS-1). The polyimide DS-1 has been synthesized by polycondensation reaction of appropriate monomers, followed by the production of fibers membranes based on the synthesized polyimide through the electrospinning procedure. The structural characterization and morphology of the electrospun polyimide fibers were investigated by FTIR, SEM and Raman, while its cytotoxicity was evaluated by the MTT assay and Live/Dead® (Molecular Probes, Eugene, OR, USA) assay. These tests were performed in contact with L929 murine fibroblasts at varying times. The high rates of viability and proliferation of cells in contact with the electrospun polyimide fibers shown by MTT and Live/Dead® tests demonstrated that this membrane is a biocompatible material. Antibacterial and anti-biofilm activities of electrospun polyimide fibers against *Staphylococcus aureus* and *Pseudomonas aeruginosa* strains were investigated.

## 2. Experimental

### 2.1. Materials

4-hydroxybenzaldehyde, aniline, aniline hydrochloride, 4,4′-oxydiphthalic anhydride (2), *N*-methyl-2-pyrrolidone (NMP), *N,N*-dimethylformamide (DMF), and dimethyl sulfoxide (DMSO), were commercially obtained from Sigma-Aldrich (Saint Louis, MO, USA) and used as received. 4,4′-diamino-4”-hydroxy triphenylmethane was obtained by the reaction of 4-hydroxybenzaldehyde with aniline in the presence of an acid catalyst, as previously reported [43]. All other reagents and solvents were commercially available and were of analytical grade. 3-(4,5-Dimethyl-2-thiazolyl)-2,5-diphenyl-2H-tetrazolium bromide (MTT), solution in PBS (5 mg/mL) were purchased from Sigma-Aldrich Chemie GmbH (Steinheim, Germany). LDH levels were quantified using the In Vitro Toxicology Assay Kit, Lactic Dehydrogenase based (Tox7 kit, Sigma-Aldrich). The Live/Dead kit was from ThermoFisher Scientific (Foster, CA, USA). The MTT solution was from Sigma-Aldrich Co, Steinheim, Germany. Bacterial strains (*Staphylococcus aureus* ATCC 25923*, Pseudomonas aeruginosa* ATCC 27853) and the L929 fibroblast cell line were purchased from ATCC (Manassas, VA, USA).

### 2.2. Synthesis of DS-1

The polyimide DS-1 was synthesized by a two-step polycondensation reaction using 4,4′-diamino-4”-hydroxytriphenylmethane and 4,4′-oxydiphthalic anhydride [33]. Yield: 89%. ATR-FTIR (cm^−1^): υ = 3057 (aromatic C–H stretching vibration); 1773 (C=O asymmetric stretching vibration); 1720 (C=O symmetric stretching vibration), 1607 and 1505 (aromatic C=C); 1368 (C–N stretching vibration); 745 (C–N ring deformation). ^1^H NMR (DMSO-d_6_, δ(ppm)): 9.35 (1H, s); 8.07–8.05 (2H, d); 7.62 (4H, s); 7.41–7.29 (8H, dd); 7.03–7 (2H, d); 6.77–6.75 (2H, d); 5.66 (1H, s).

### 2.3. Fabrication of Electrospun Polyimide Fibers

The polyimide (23% w/v) solution in DMF was fed from a 1 mL plastic syringe equipped with a 21-gauge stainless steel needle. The electric potential, obtained from a dual power source, was fixed at a positive output of +10 kV and a negative output of −2 kV. The solution feed rate was set to 5 µL/min. The electrospun polyimide fibers were collected on an anti-adhesive paper foil. The solution was electrospun at room temperature (23 ± 1 °C). The collected electrospun polyimide fibers were allowed to dry under vacuum oven at 37 °C [44].

### 2.4. Characterization and Methods

FTIR spectrum of electrospun polyimide fibers was obtained by using BioRad ‘FTS 135′ FTIR spectrometer equipped with a Specac “Golden Gate” ATR accessory. A LUMOS Microscope Fourier Transform Infrared (FTIR) spectrophotometer (Bruker Optik GmbH, Ettlingen, Germany), equipped with an attenuated total reflection (ATR) device was used to record the scans between 4000 and 500 cm^−1^ at a resolution of 4 cm^−1^.

^1^H NMR spectrum was obtained on a Bruker Advance DRX 400 spectrometer, equipped with a 5 mm, direct detection, multinuclear probe, operating at 400.1 MHz. The polymer sample was dissolved in DMSO and then measured at room temperature. ^1^H chemical shifts (δ) were quoted in ppm relative to the residual peak of the solvent (ref. ^1^H: DMSO-*d_6_*: 2.51 ppm).

The morphological characteristics of their surfaces were studied using a scanning electron microscope type Quanta 200 (FEI, Eindhoven, Netherland), operating at 20 kV with secondary electrons in low vacuum mode, the magnification being indicated on each micrograph. The SEM studies were performed on uncoated samples fixed on aluminium substrate. The average fibers diameter was calculated from 42 fibers in three randomly selected areas using Scandium software on the SEM micrographs of the samples.

Raman spectra of electrospun polyimide fibers were recorded using an inVia™ confocal Raman microscope spectrometer (Renishaw plc, Gloucestershire, UK) equipped with a 633 nm excitation laser line and a Leica DM2700 microscope with 5×, 20× and 100× objectives and a Deep Depletion Renishaw CCD Centrus array detector. Scans were accumulated to obtain the spectra in the range of 100–4000 cm^−1^ with a resolution of 1 cm^−1^ and a laser power of 17 mW. Electrospun polyimide fibers were measured directly from metal discs.

Water uptake: To measure the water uptake, specimens of electrospun fibers (small square pieces 1 cm × 1 cm) were cut. These samples were immersed in 10 mL of distilled water, for different periods of time, from 10 min to 24 h. Then, the samples were taken out and the excess surface water was quickly removed by blotting paper. The weight of the samples in wet state at each time point was noted using an electrical balance immediately. The degree of water uptake for every swelling period was calculated using the following formula:

*WU*_1–13_(%) = *Wwet*_1–13_ − *Wdry*/*Wdry* × 100 (1)

where, *Wwet*_1–13_ is the wet weight of electrospun polyimide fibers at every swelling period *t*_1_ to *t*_13_, *Wdry* is the dry weight of electrospun polyimide fibers.

Static contact angle measurements were performed using a CAM 101 (KSV Instruments, Helsinki, Finland) system, equipped with a liquid dispenser, video camera, and drop shape analysis software. In order to obtain the statistical results, the experiments were conducted using double-distilled water on three different regions selected for the surface of the samples. All the measurements were performed at room temperature.

The morphological stability behavior was evaluated from the morphological change of the electrospun polyimide fibers. In this regard, the dried electrospun polyimide fibers were cut into small square pieces (1 cm × 1 cm). Each cut of electrospun polyimide fibers membrane was exactly measured for initial weight and then immersed in 10 mL of phosphate buffer solution (PBS, pH 7.4 at 37 °C) for a period of two days and six days, respectively. When the predetermined time expired (two and six days), each sample of electrospun polyimide fibers membrane was extracted from vials, rinsed several times with distilled water to remove residual buffer salts, and dried to constant weight in an oven at 60 °C. The morphological change was estimated from the SEM observation as mentioned above, after immersion in PBS for two and six days, respectively.

Evaluation of the anti-biofilm effect of the electrospun-polyimide fibers membrane was performed following a protocol reported in the literature [45], as follows: *Staphylococcus aureus* ATCC 25923 and *Pseudomonas aeruginosa* ATCC 27853 strains were cultured on tryptic soy agar (Thermo Fisher Scientific, Waltham, MA, USA) for 18 h prior to the experiment. Subsequently, one colony was resuspended in 3 mL of tryptic soy broth media (MP Biomedicals, Solon, OH, USA) followed by incubation at 37 °C (18 h, 200 rpm). The suspension was adjusted to an optical density of 0.52 (at OD 562 nm) which corresponds to 10^9^ colony forming units (CFU) per mL. The inoculum was further diluted to 10^6^ CFU/mL in simulated body fluid supplemented with 10% fetal bovine serum. The samples for testing-tissue culture plastic control (TCPS), DS-1 and, silicone cathether were plated onto the wells of a 24-well plate. Next, 1 mL of the 10^6^ CFU/mL solution of *P. aeruginosa/ S. aureus* in simulated body fluid was pipetted into each well, while ensuring complete submersion of the samples. Samples were kept at 37 °C at 5% carbon dioxide to allow biofilm formation. After 24 h, excess bacteria were aspirated, and the samples were thoroughly washed with PBS (three times) to ensure the removal of all media residue and non-adherent bacteria. Samples were removed from the wells with a sterile forcep and immersed in 1 mL of PBS. To strip all adherent bacteria from the sample into solution, the tubes were vortexed at 3000 rpm (20 min). The resulting suspension was serially diluted in PBS in order to quantify the CFU/mL values.

DS-1 biocompatibility tests were performed during one week of culture in standard conditions, by analyzing cell behavior in response to DS-1 materials. Fibroblasts from L929 cell line (ATCC, CCL-1, ATCC, Manassas, VA, USA) were cultured in contact with DS-1 electrospun polyimide fibers at a density of 2 × 10^4^ cells/cm^2^ and monitored for 24 h to allow cell adhesion and forming of cell-material constructs. For biocompatibility assessment, these constructs were permanently compared to L929 culture on TCPS, which was used as a control. Biocompatibility evaluation comprised of viability and proliferation analysis via MTT test, DS-1 cytotoxicity analysis by LDH assay, and visualization of live and dead cells in the culture by Live/Dead assay.

MTT assay is a quantitative method which allows evaluation of cells’ metabolic activity. Briefly, L929 murine fibroblasts were incubated with 1 mg/mL [3-(4.5-dimethylthiazol-2yl)]-2.5-diphenyltetrazolium bromide (MTT) solution for 4 h in the dark, at 37 °C. After incubation, formazan crystals were solubilized with isopropanol, resulting in purple solution, quantified by spectrophotometry at 550 nm, using FlexStation3 (Molecular Devices, Foster City, CA, USA).

Electrospun polyimide fibers’ cytotoxicity exerted on the cells was investigated using LDH test (Tox7 kit, Sigma-Aldrich), according to manufacturer’s instructions—cells that no longer have membrane integrity release lactate dehydrogenase (LDH) into the culture medium. In our experiment, the medium was collected and mixed with the kit’s components in order to be evaluated after two and six days of culture by spectrophotometric measurement at 490 nm.

Live/Dead assay is a qualitative test, based on two components revealing simultaneously stain live and dead cells and allowing visualization of cell dispersion and morphology inside the materials. The test was assessed following manufacturer’s protocol using Live/Dead kit (ThermoFisher Scientific, Foster City, CA, USA). The kit includes calcein AM—staining cells that have an intact cell membrane (live cells) in green and ethidium bromide—staining cells with a damaged membrane integrity (dead cells) in red. Images were obtained using confocal microscopy (Carl Zeiss LSM 710, Jena, Germany) and then processed with Zeiss Zen 2010 software.

Statistical analysis was performed using Graph Pad Prism 6.0 software, One-Way ANOVA and Bonferroni correction. Statistically significant values were considered for *p* < 0.05.

## 3. Results and Discussion

**Scheme 1:**
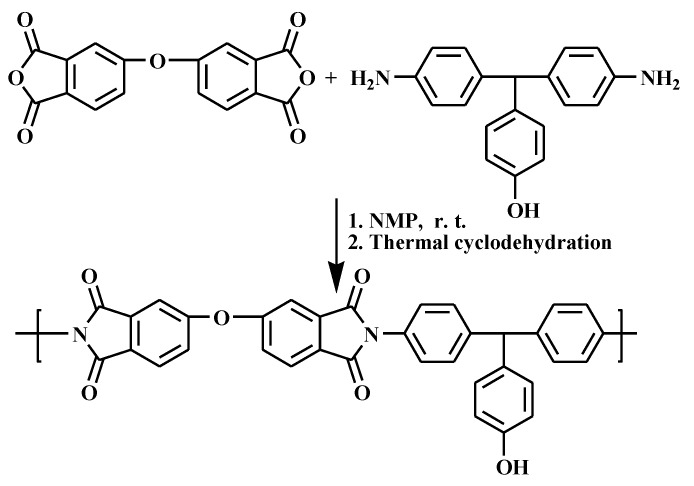
Schematic pathway to describe the synthesis of polyimide DS-1.

The chosen DS-1 polyimide have been synthesized by the classic two steps polycondensation reaction, using equimolecular amounts of 4,4′-diamino-4”-hydroxytriphenylmethane and 4,4′-oxydiphthalic anhydride as it was depicted in Scheme 1, following a detailed method described elsewhere [33]. The synthesized DS-1 polyimide was soluble in common organic solvent such as dimethyl sulfoxide (DMSO), *N,N*-dimethylformamide (DMF), *N,N*-dimethylacetamide (DMAc), and *N*-methyl-2-pyrrolidone (NMP). The good solubility of DS-1 was attributed to the presence of the pendant phenol groups in the structural unit, which reduced the inter-chain interactions and thus increasing the solubility. The chemical structure of DS-1 polyimide was confirmed by the ATR-FTIR and ^1^H NMR spectroscopy. The complete imidization process was confirmed by the presence, in the FTIR spectrum, of imide characteristic absorption bands located at 1773 cm^−1,^ attributed to C=O asymmetric stretching vibration, 1720 cm^−1^, attributed to C=O symmetric stretching vibration, and 745 cm^−1^, attributed to C–N ring deformation. Moreover, the chemical structure of DS-1 polyimide was confirmed by ^1^H NMR, with complete assignment of the protons as it was listed in the experimental section.

**Figure 1:**
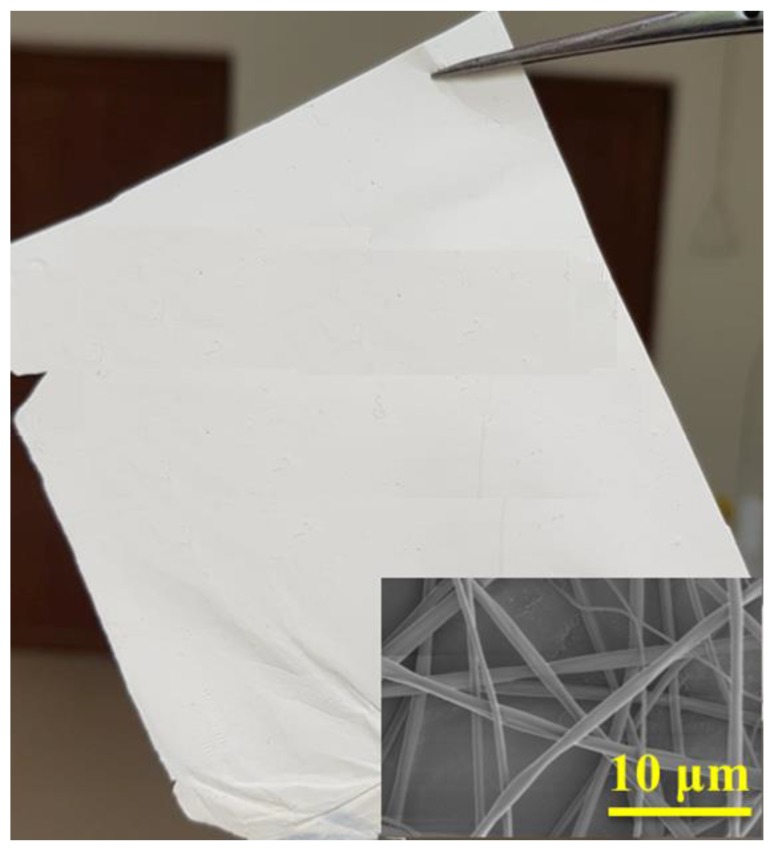
Image showing large scale membrane and SEM image of the electrospun polyimide fibers obtained by electrospinning process.

Further in this study, we performed cytotoxicity tests on a membrane based on electrospun polyimide fibers, obtained from DS-1 polyimide, developed for various biomedical applications, for example: wound dressing, pharmaceutical patches, bandages or meshes, etc. In this respect, facile production of hydroxy-functional electrospun polyimide fibers, through electrospinning process, is described further. All electrospinning parameters such as polymer concentration, solvents, flow rate, the distance between drum and jet were optimized according to our previously reported work [44]. Thus, following the rationale optimization procedure of electrospinning process, largely described in the above cited paper, the optimal condition to obtain DS-1 polyimide fibers having small diameters and narrow distribution without beads were found to be: 23 wt%, +10 kV/−2 kV dual voltage, 10 cm spinning distance at 5 µL/min feed rate. Using these parameters, the average fibers diameters for the electrospun polyimide fibers was found to be equal with 730 ± 89 nm. As shown in Figure 1, uniform, smooth, and bead free fibers were produced.

**Figure 2:**
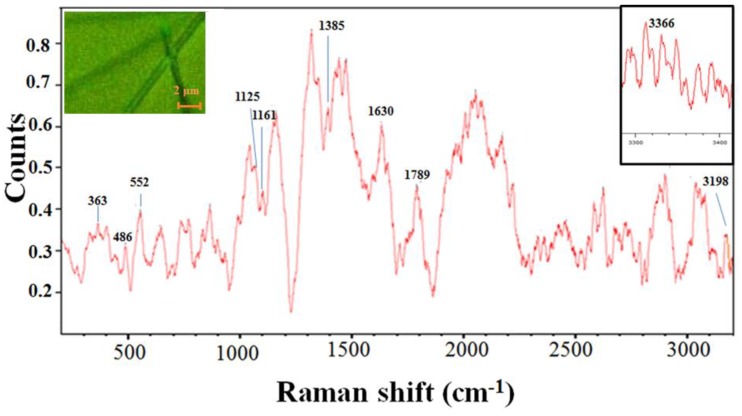
Raman spectrum and Raman image (left corner up inset) obtained by Raman spectroscopy measurements on the DS-1 electrospun polyimide fibers.

Reference Raman spectra obtained from electrospun polyimide fibers contained several distinctive bands. Figure 2 shows the averaged reference Raman of electrospun polyimide fibers while the left corner up inset display 50× optical image of electrospun polyimide fibers. Raman vibration spectroscopies were taken to assess the nature of chemical bonding, interactions, conformations, and even orientations of molecules in electrospun polyimide fibers. In the high-wavenumber spectral region (3050–3500 cm^−1^) of the Raman spectrum, two low-intensity bands attributed to the O-H stretching modes are present at 3366 and 3198 cm^−1^. Characteristic bands for polyimide are imide I (C=O stretch at 1789 cm^−1^), imide II (C–N–C axial vibration stretch at 1385 cm^−1^), imide III (C–N–C transverse vibration stretch at 1125 cm^−1^). These detected functional bands indicated that the electrospun polyimide fibers were highly imidized under the curing conditions used. The bands near 1630 cm^−1^ and 1161 cm^−1^ were attributed to ring stretching modes of benzene rings in the polyimide backbone. The bands at 363, 486, and 552 cm^−1^ can be attributed to C–O–C stretching. The morphology of electrospun polyimide fibers was confirmed by Raman spectroscopy (Figure 2, left corner up inset).

It has been noticed in the literature that polyimides are generally biocompatible [26,46,47,48]. However, freestanding and flexible polyimide films or membranes suffer from the point of view of interaction properties of the polymer substrate with the cells. These interactions are essential for targeted biomedical applications such as transdermal therapy, bioactive wound dressing, or dermal repair/regeneration. Some surface modification processes were designed to incorporate certain functional groups, e.g., amino, hydroxyl or methyl, in order to influence the non-specific interactions of the protein with the surface and thereby influence cellular interaction. Thus, our interest here was not only to provide a polyimide substrate in the form of an electrospun fibers membrane, but also, to design a chemical structure for the chosen polyimide in such manner that it contains free functional hydroxyl groups, without altering the substrate by surface modification procedures.

It is generally known that surface wettability is an important parameter of membranes for both fundamental and practical applications [38]. Surface wettability influences biological response of the biomaterials for application in biomedicine. For example, wettability can be responsible for some capabilities of wound dressing materials, such as the ability to absorb exudates, the regulations of moist environment, and stimulation of the cell regeneration rate [49]. The role of wettability in protein adsorption, blood coagulation, cell and bacterial adhesion, etc. has been demonstrated in several studies [50,51,52]. Moreover, the dynamics of the surface of a material can be described by means of contact angle measurements because the surface layer is examined at a depth comparable to the radius of action of the interfacial forces involved in the surface restructuring [53].

**Figure 3:**
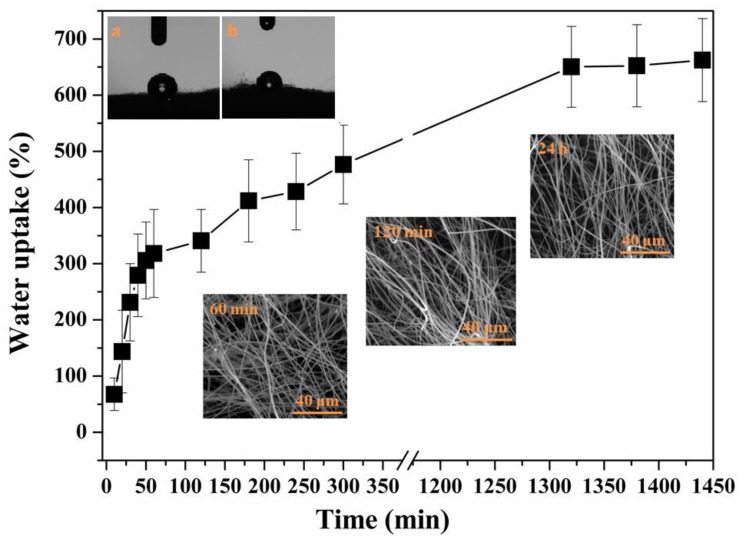
Water uptake capacity of DS-1 fibers, and SEM images of the samples after swelling periods of 60 min, 120 min and 24 h, respectively. In the left corner up insets, images from the contact angle experiments on the sample before swelling (**a**) and after swelling study (**b**), are given.

To investigate the effect of the presence of free hydroxyl functional groups on wettability properties of DS-1 polyimide, water contact angle and water uptake capacity of DS-1 fibers membrane were measured with obtained results being presented in Figure 3. The water contact angle of the electrospun polyimide fibers was > 90° (Figure 3a, left corner up inset), the value of 108.79 ± 0.5^o^ was indicative of a typical hydrophobic feature. In general, it has been shown in the literature that surface wettability of electrospun fiber materials is associated with a variety of factors, which include the nature of the polymeric material, and some characteristics related to the structure of the surface at nano-micro level, such as roughness, fiber diameter, pore size, and porosity [54]. One reason for hydrophobic behavior of DS-1 electrospun fibers could be explained by its varying microstructure and large specific surface. However, there was a reasonable drop in the water contact angle for DS-1 fibers membrane, in comparison with other polyimide structures reported in the literature [53] reflecting the advantage of the presence of OH groups along the DS-1 macromolecule [54].

To act as a promising wound dressing material, an electrospun fibers membrane should display exudate soaking ability, wound wetting capacity, non-adherence, easy to remove ability [55]. The properties can be determined through water uptake capacity. In this study, the absorption capability of DS-1 polyimide fibers membrane was studied at different time points from 10 min to 24 h. The DS-1 membrane was found to have an impressive absorption percentage of 620% after 24 h of incubation. This significant enhancement can be attributed to the availability of OH functional groups. Moreover, the porous nature of DS-1 fibrous membrane may also have contributed to the equilibrium absorption percentage by supporting the diffusion of more water molecules into the fiber network. In order to test the dimensional stability of DS-1 fibers after swelling experiments, the dry samples corresponding to 60 min, 120 min, and 24 h of swelling were subjected to SEM investigation. As it can be observed in Figure 3, all the images, corresponding to three different time periods of swelling, exhibited similar fibrous morphology for the studied samples, as the non-swollen DS-1 fibers. To complement these observations, contact angle measurements were performed on the sample subjected to the swelling experiments. After 24 h of swelling, the sample was allowed to dry, then, its contact angle value was measured. Unfortunately, the planarity of the membrane is a little bit affected after the swelling process, thus, the new contact angle increased slightly (112.79 ± 0.23°, Figure 3b, left corner up inset), probably due to the modification of surface topography.

The swelling absorption results indicated that the currently studied electrospun fiber membrane, based on polyimide having free –OH functional groups, provided broader hydrophilic sites inside the polymer matrix and higher surface area for water absorption. Thus, from practical point of view, this would not only prevent the loss of the fluids and nutrients from the human body in the in vivo tests, but would also increase the cell attachment and proliferation rate of fibrous polyimide membrane [56], which is advantageous for targeted specific applications such as bioactive wound dressing material, medical/pharmaceutical patches or bandages and other.

**Figure 4:**
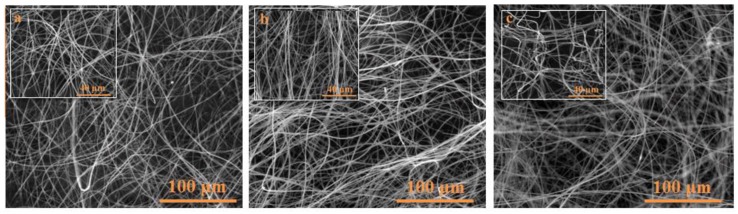
SEM micrographs of the electrospun polyimide fibers (**a**), electrospun polyimide fibers exposed to PBS (Phosphate-Buffered Saline) for two days (**b**) and six days (**c**) at 100 μm magnification and 40 μm, respectively.

The physicochemical stability of the studied DS-1 polymeric matrix is an important parameter when looking for potential biomedical applications of this material. In order to check if the morphology and the integrity of the structure of electrospun polyimide fibers could be affected by the PBS solution, small pieces of DS-1 samples (1 cm × 1 cm) were immersed in the PBS solution for a period of two and six days, respectively. After washing and drying these samples they were subjected to SEM investigation (Figure 4). As it has can be seen in the images taken at 100 μm magnification the electrospun polyimide fibers keep the morphology of their fibrous structure after immersing the samples in PBS either for two or six days, respectively. The average diameter of the electrospun polyimide fibers was calculated and it was observed that the average diameter was not affected by the presence of PBS solution (730 ± 89 nm) (the insets micrographs at 40 μm magnification). In order to complement these findings, after performing the swelling experiments, the samples were allowed to dry in an oven for 36 h at 60 °C, then, they were weighed to confirm their initial masses, as a parameter for the morphological stability, and structural integrity of the DS-1 fibers membrane.

**Figure 5:**
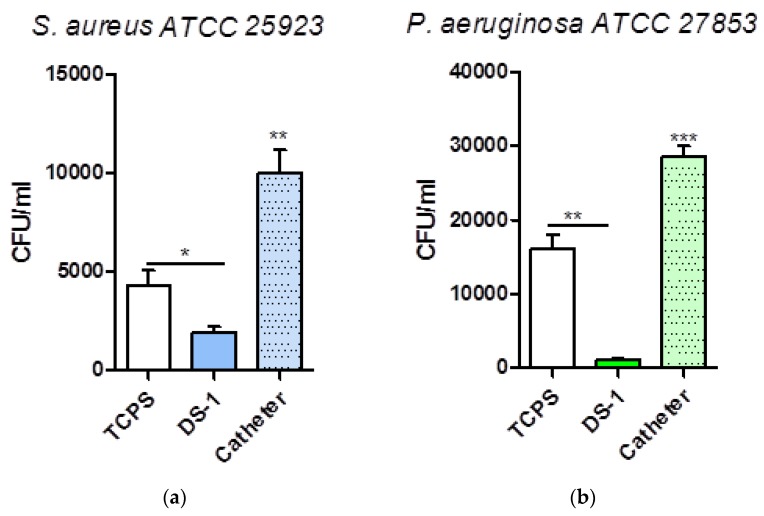
Strains adherence of *S. aureus* and *P. aeruginosa* (ATCC strains) to electrospun polyimide fibers. (**a**) note; (**b**) note.

In the next step of our study, the antibiofilm potential of electrospun polyimide fibers was examined against biofilm producing bacteria. We investigated the adherence of *S. aureus* and *P. aeruginosa* ATCC strains to electrospun polyimide fibers. Figure 5 demonstrates the very good antibiofilm properties of electrospun polyimide fibers compared to the controls (represented by tissue culture plastic control as well as urinary catheter). The antimicrobial behavior of electrospun polyimide fibers is more pronounced in the case of *S. aureus* compared to *P. aeruginosa* and it can be explained considering the presence of the pendant phenolic OH groups in the polyimide structure.

We chose a urinary catheter as positive control because when used in a clinical setting, it connects the heavily colonized perineum with the normally sterile bladder and therefore provides an entry route for microorganism multiplication and biofilm formation. *S. aureus* as well as *P. aeruginosa* produce on urinary catheters biofilms hard to eradicate in clinical practice. In our experiment, urinary catheters were colonized in 24 h by 3 × 10^5^ CFUs of *P. aeruginosa* and 10^5^ CFUs of *S. aureus*. Compared to the urinary catheter control, the novel polyimide electrospun fibers membrane developed within this study showed a two-log reduction in bacterial growth for both *S. aureus* and *P. aeruginosa* (*P < 0.05, **P < 0.01, **P < 0.001, n = 3, ±S.E.M). In addition, DS-1 material proved to have an enhanced antibiofilm activity compared to the TCPS control (*p* = 0.0375 for *S. aureus*, *p* = 0.0016 for *P. aeruginosa*).

**Figure 6:**
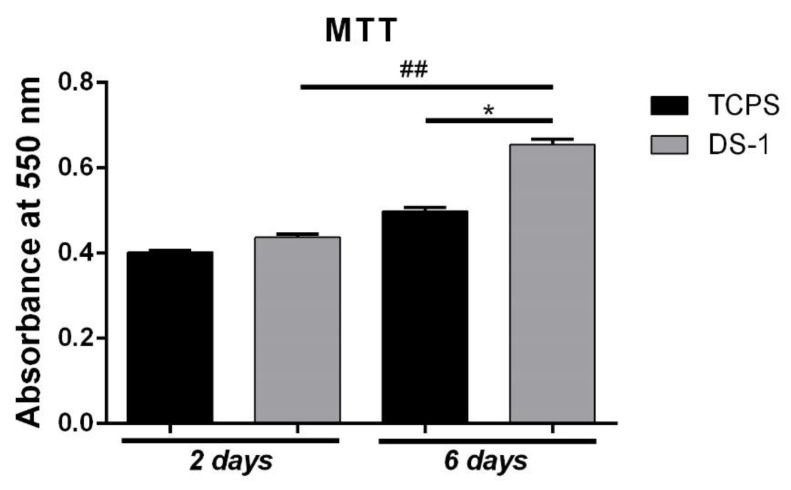
Cell viability and proliferation profile registered for DS-1 after two and six days of culture, evaluated by MTT assay. Statistical significance: * *p* < 0.05 (DS-1 six days versus TCPS six days); ## *p* < 0.01 (DS-1 six days versus DS-1 two days).

MTT assay results, after two days of culture in standard conditions, showed an overall good viability of L929 cultured in contact with both control system (CTRL) and the DS-1 material. The DS-1 material indicated a slightly increased viability (Figure 6) compared to the TCPS control value, although no statistical significance was observed. After the cells were maintained in culture for six days with the electrospun polyimide fibers, the higher viability tendency observed at two days became obvious. Hence, the highest statistically significant (*p* < 0.05) increased viability rate was noticed for the cells cultured in contact with the DS-1 composite in comparison to the control system. The results also indicated a statistically significant (*p* < 0.01) increased cell proliferation after six days of culture on DS-1. Since viability and proliferation were better on the DS-1 material than on TCPS control, this might suggest that the electrospun polyimide fibers membrane did not affect the fibroblasts cell viability in the evaluated time points, probably due to material’s high surface area to volume ratio. 

**Figure 7:**
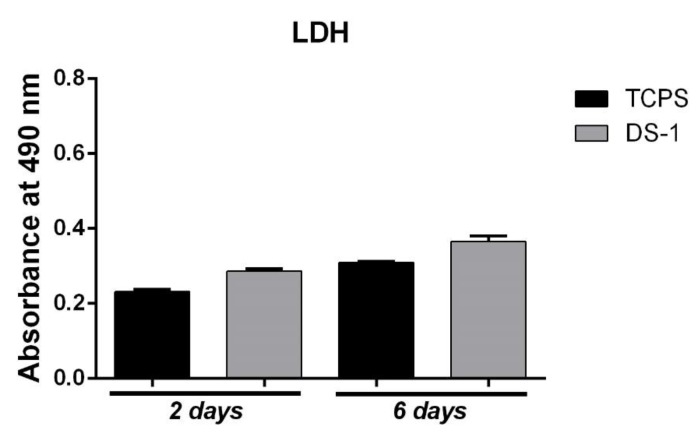
Cytotoxic level registered for DS-1 after two and six days of culture, evaluated by LDH assay.

After two days of culture in standard conditions, LDH assay indicated a similar cytotoxic effect (Figure 7) for DS-1 material as for TCPS, since a low number of dead cells were found after they were kept in contact with the materials. At six days, the cytotoxicity of the DS-1 composite slightly increased, but no statistical significance was observed between its cytotoxicity and the one of TCPS control. The results suggest that electrospun polyimide fibers did not exert a significant cytotoxic effect on the cellular component.

**Figure 8:**
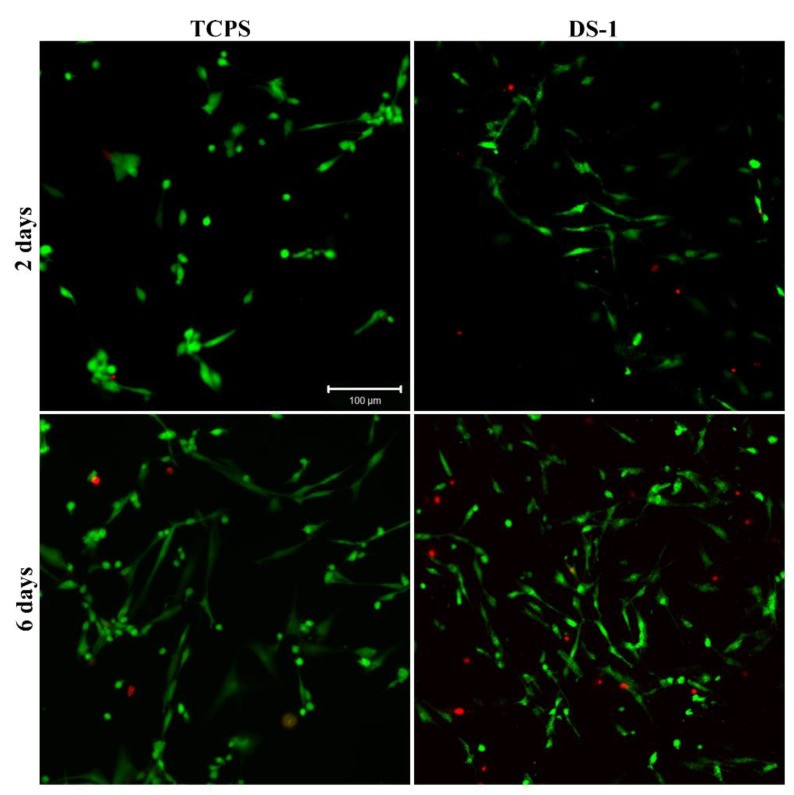
Confocal microscopy of live (green) and dead (red) cells cultured in contact with DS-1 for two and six days.

Live/Dead staining supports the results of the MTT and LDH assays, given that the number of viable cells was considerably higher than the number of dead cells. The number of live cells was elevated on the DS-1 material compared to the control, after two days of culture in standard conditions. Six days after cell seeding, a great number of viable cells was observed on the DS-1 material in comparison to TCPS. Furthermore, confocal microscopy allowed the visualization of their tendency to proliferate on the electrospun polyimide fibers (Figure 8). These results could be due to the extremely interconnected pore network and thin fiber diameters in the fibers material that mimic the extracellular matrix environment.

## 4. Conclusions

The current study presents the preparation of electrospun fibers membrane based on a polyimide carrying hydroxyl functional groups. Structure-properties correlations related to water uptake capacity and water contact angle have been made. The limitation of the relatively high value of the contact angle could be overcome by further design of the chemical structure of the chosen polyimide. The cytotoxicity of the prepared membrane, evaluated by the MTT assay and Live/Dead^®^ (Molecular Probes) assay, revealed high rates of viability and proliferation of fibroblast cells in contact with the electrospun polyimide fibers. Antibacterial and anti-biofilm activities of electrospun polyimide fibers against *Staphylococcus aureus* and *Pseudomonas aeruginosa* strains were investigated, resulting in aggregation of bacteria, and a reduction in biofilm development. According to LDH assay, after two days of culture in standard conditions, a high proportion of live cells has been observed on the tested samples and this proportion was even higher after six days of culture. All the obtained results imply further studies on this type of materials in order to establish their mechanical properties and to target specific biomedical application, such as wound dressing material, medical/pharmaceutical patches or bandages etc., at the interface between the material and the cells or living tissues.
